# Supplementary material for: Comparative Analysis of Biological Signatures between Freshly Preserved and Cryo-Preserved Bone Marrow Mesenchymal Stem Cells
Source: Cells. 2023 Sep 26;12(19):2355. doi: 10.3390/cells12192355 (PMC10571833; doi:10.3390/cells12192355)
Supplement: Supplementary file 1 [file cells-12-02355-s001.zip › cells-2594427-supplementary.pdf]

Comparative Analysis of Biological Signatures between Freshly Preserved and Cryo-Preserved Bone Marrow Mesenchymal Stem Cells
(Supplementary Materials)

Taesic Lee [1,2,†], Sangwon Hwang [3,†], Dongmin Seo [4], Sungyoon Cho [5], Sunja Yang [5], Hyunsoo Kim [5], Jangyoung Kim [6] and Young Uh [7,*]

1 Division of Data Mining and Computational Biology, Regenerative Medicine Research Center, Wonju Severance Christian Hospital, Wonju 26426, Republic of Korea; ddasic123@yonsei.ac.kr
2 Department of Family Medicine, Yonsei University Wonju College of Medicine, Wonju 26426, Republic of Korea
3 Department of Precision Medicine, Yonsei University Wonju College of Medicine, Wonju 26426, Republic of Korea; arsenal@yonsei.ac.kr
4 Department of Medical Information, Yonsei University Wonju College of Medicine, Wonju 26426, Republic of Korea; dmseo@yonsei.ac.kr
5 Pharmicell Co., Ltd., Seongnam 13229, Republic of Korea; sungyoon9030@pharmicell.com (S.C.); sun-ja@pharmicell.com (S.Y.); khsmd@pharmicell.com (H.K.)
6 Department of Internal Medicine, Yonsei University Wonju College of Medicine, Wonju 26426, Republic of Korea; kimjy@yonsei.ac.kr
7 Department of Laboratory Medicine, Yonsei University Wonju College of Medicine, Wonju 26426, Republic of Korea
* Correspondence: u931018@yonsei.ac.kr; Tel.: +82-33-741-1592
† These authors contributed equally to this work.

Supplementary Figure S1. Actual photographs for manufacturing MSC.

**Culturing of MSC**		
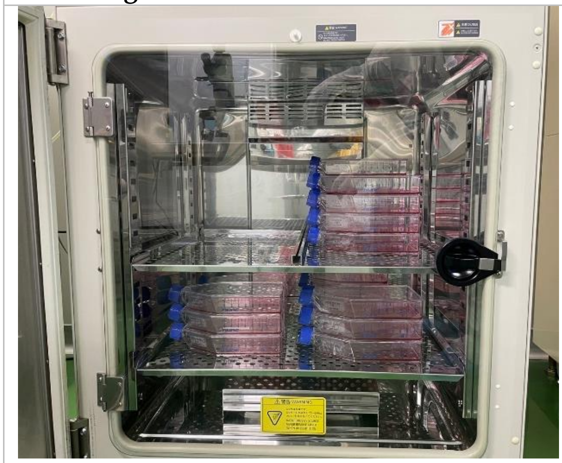	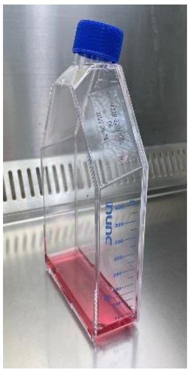	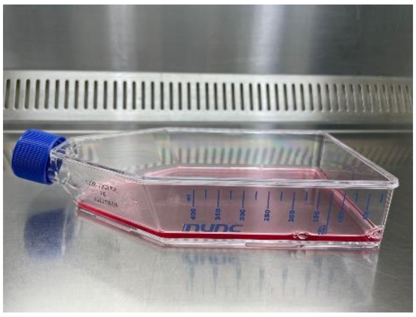

**Before freezing**	**After freezing**
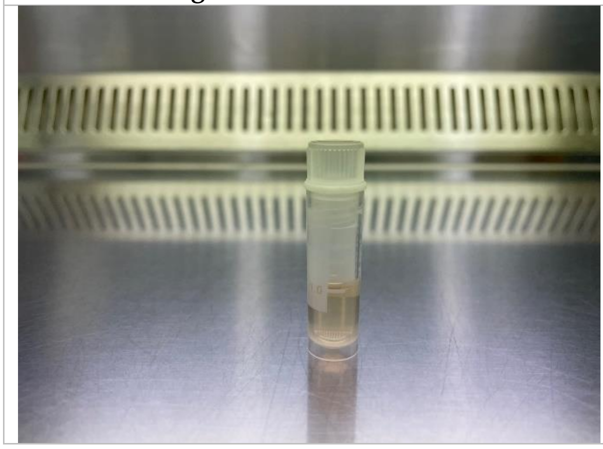	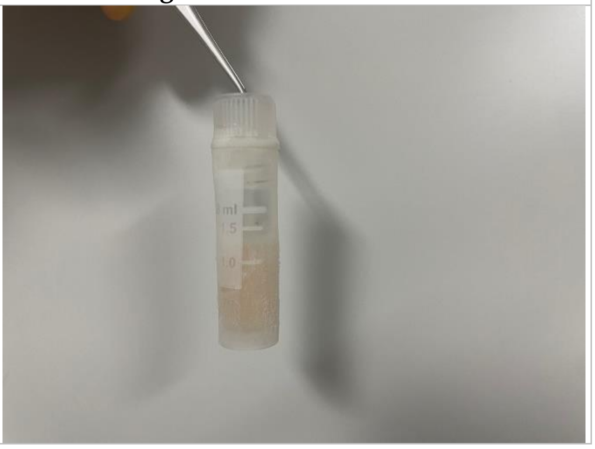

Supplementary Figure S2. Distribution of the amount of BM extracted.

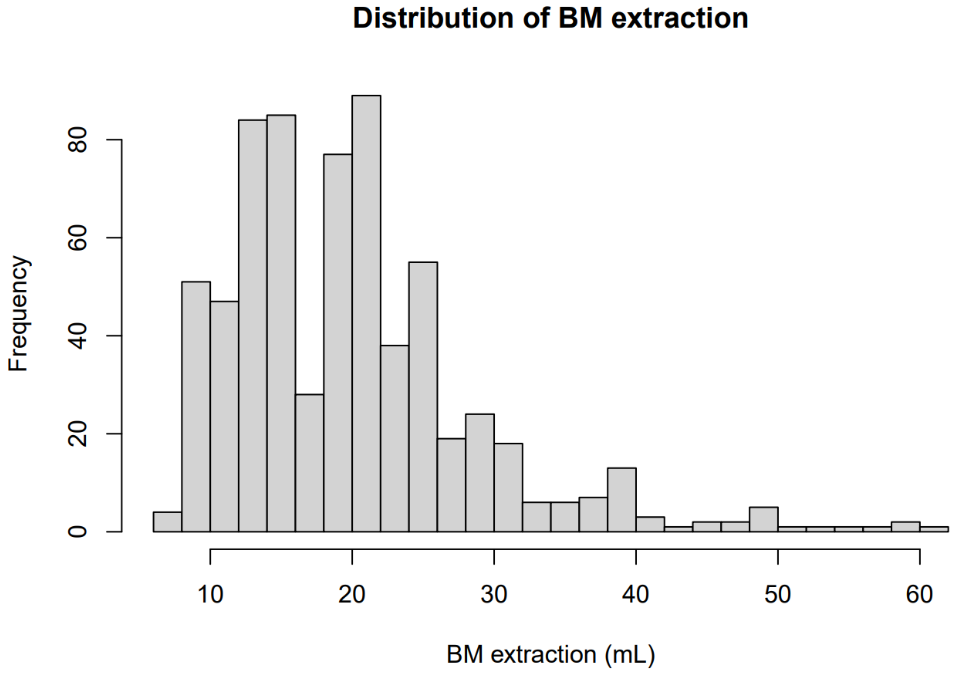

Supplementary Figure S3 Distribution of the number of MNCs at each passage according to the type of preservation.

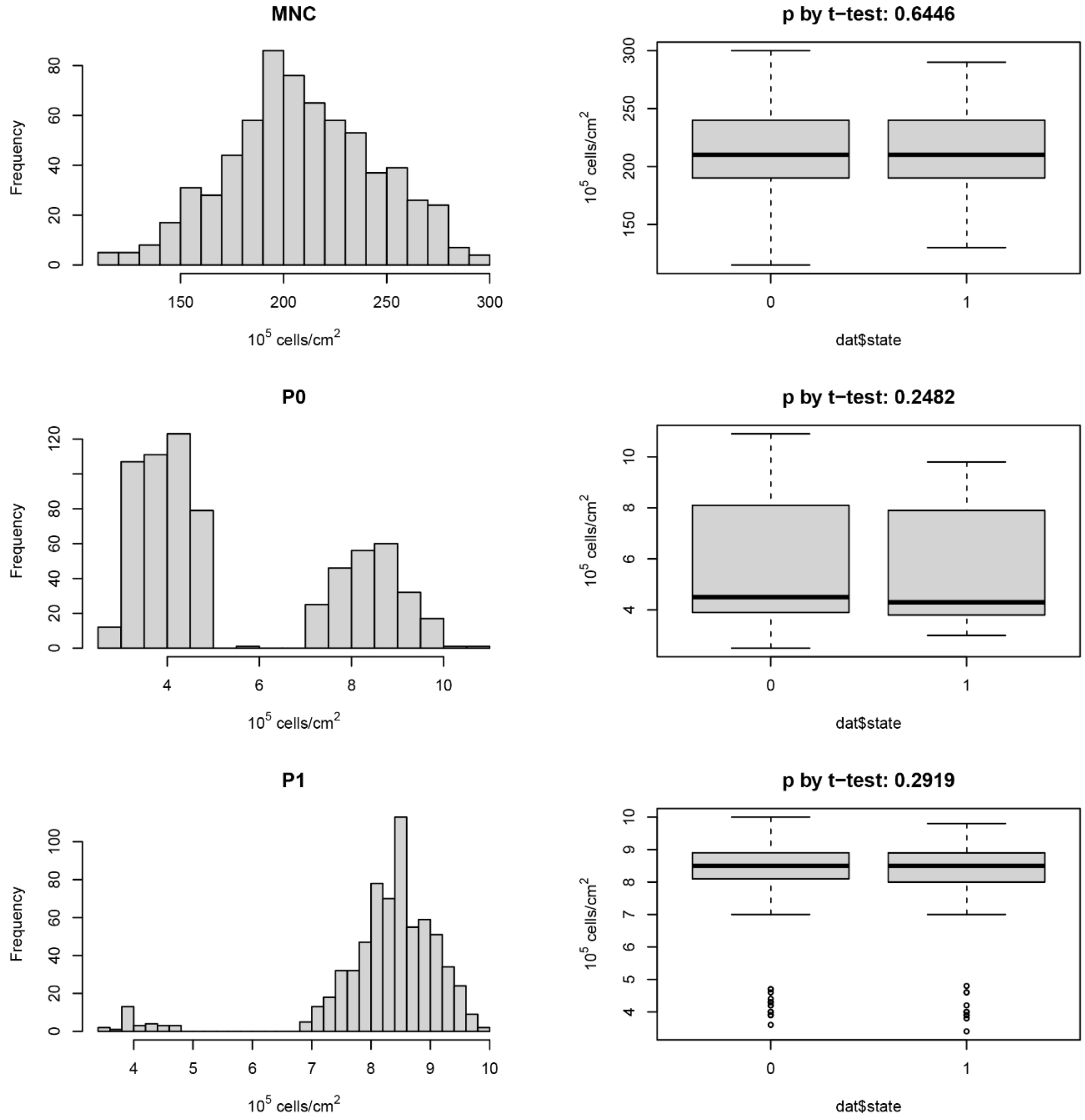

Supplementary Figure S3. Distribution of the number of MNCs at each passage according to the type of preservation, continued.

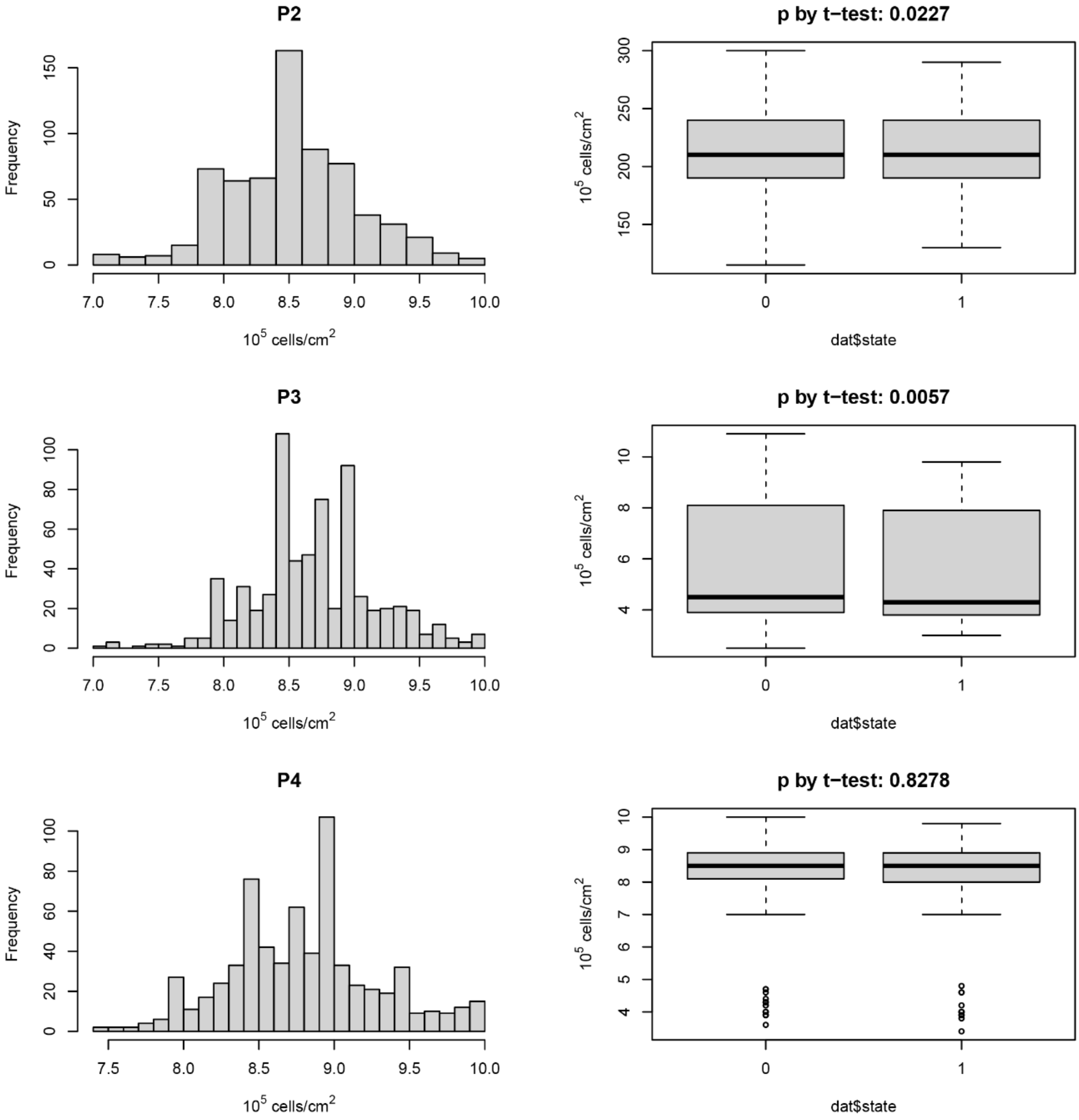

Supplementary Figure S4 . MSC signatures abstracted with the PCA method.

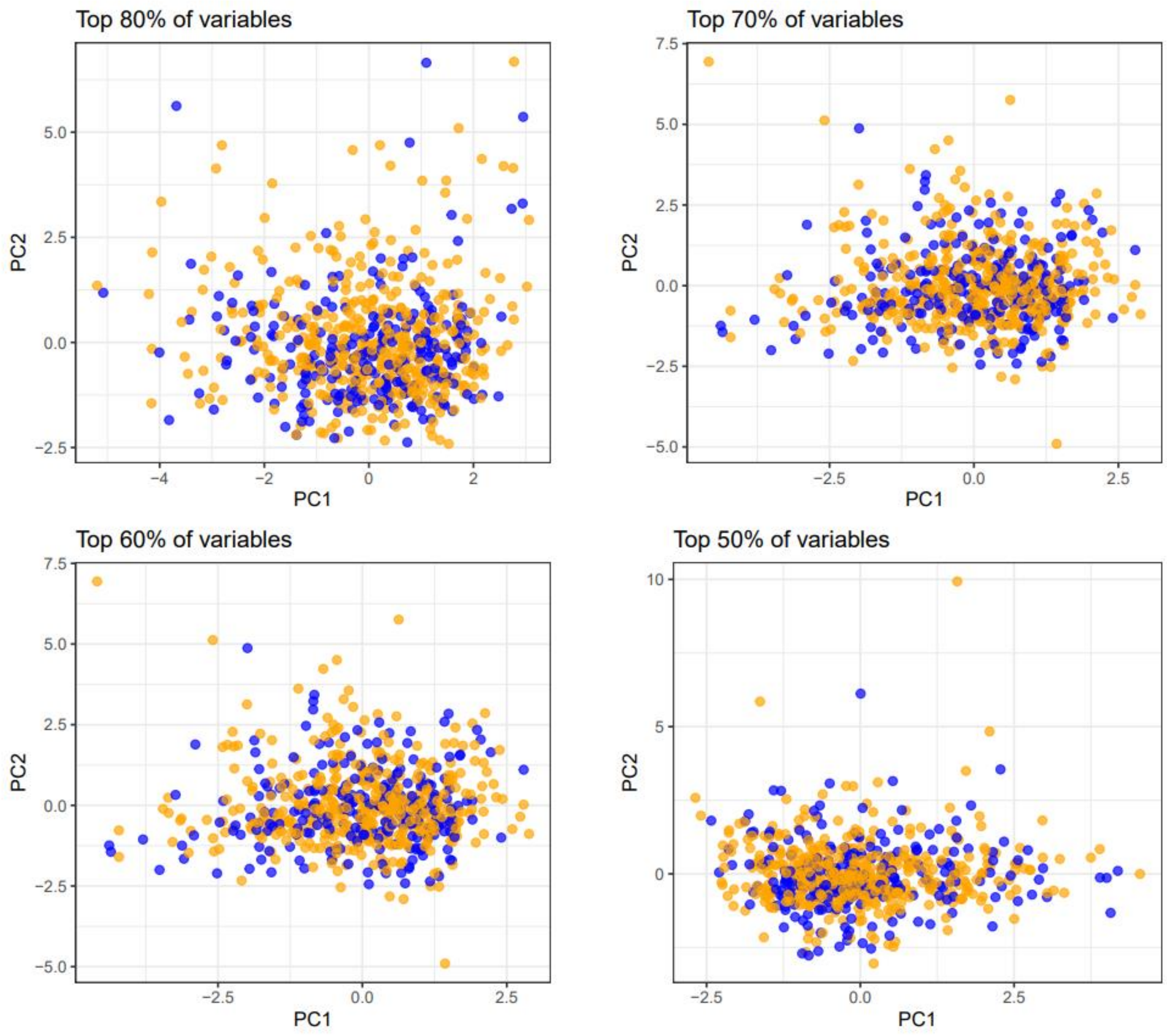

Supplementary Table S1. General characteristics of BM-MSCs according to the method of preservation.

Features	Freshly preserved	Cryo-preserved	*p*-value
**Number**	426	245	
**Age, years**	58.5 ± 0.78	55 ± 1.34	0.024
**Manufacturing duration, days**	28.3 ± 0.11	28.2 ± 0.12	0.726
**BM amount, mL**	22.8 ± 0.43	16.1 ± 0.41	<0.001
**MNC, 10^5 cells/cm^2**	213.2 ± 1.82	211.9 ± 2.14	0.645
**MSC at P0, 10^5 cells/cm^2**	5.7 ± 0.11	5.4 ± 0.14	0.248
**MSC at P1, 10^5 cells/cm^2**	8.3 ± 0.05	8.2 ± 0.07	0.292
**MSC at P2, 10^5 cells/cm^2**	8.6 ± 0.02	8.5 ± 0.03	0.023
**MSC at P3, 10^5 cells/cm^2**	8.8 ± 0.02	8.7 ± 0.03	0.006
**MSC at P4, 10^5 cells/cm^2**	8.8 ± 0.02	8.8 ± 0.03	0.828

Continuous variables are expressed as mean ± standard deviation*p*-value was evaluated using Student's t-test. Abbreviations: BM, bone marrow; MNC, mononuclear cell; MSC, mesenchymal stem cell; P, passage in cell culture

Supplementary Table S 2 . Summary statistics (interquartile ranges and median) for PDT.

	25 percentiles	Median	75 percentiles
**P0_doubling time**	50.26	59.19	71.61
**P1_doubling time**	49.72	59.09	70.105
**P2_doubling time**	55.34	64.13	74.055
**P3_doubling time**	54.87	65.71	78.705
**P4_doubling time**	70.72	84.58	106.98

Supplementary Table S3. Summary statistics (interquartile ranges and median) for MSC viability.

	**25 percentiles**	**Median**	**75 percentiles**
**P0_viability**	97.6	98.5	99.2
**P1_viability**	97.5	98.6	99.3
**P2_viability**	98	98.7	99.3
**P3_viability**	97.5	98.6	99.2
**P4_viability**	86.65	90.7	96.2

Supplementary Table S4 . Summary statistics (interquartile ranges and median) for CD markers.

	25 percentiles	Median	75 percentiles
**CD105**	98.335	99.36	99.76
**CD73**	99.73	99.91	99.97
**CD34**	0.63	0.77	0.92
**CD45**	0.56	0.73	0.88
**CD14**	0.81	0.99	1.21

Supplementary Table S5 . Summary statistics (interquartile ranges and median) for CD markers.

	**25 percentiles**	**Median**	**75 percentiles**
**VEGF**	1206.05	1537.8	2071.55
**MCP.1**	245.3	394	587.4
**IL.6**	1090.7	1664.8	2442.2
